# Empathic Dimensions Influence Motor Resonance Magnitude During Transitive but Not Intransitive Action Observation: A Retrospective Investigation

**DOI:** 10.3390/brainsci15111174

**Published:** 2025-10-30

**Authors:** Giacomo Guidali, Maria Franca, Eleonora Arrigoni, Michela Picardi, Alberto Pisoni, Nadia Bolognini

**Affiliations:** 1 Department of Psychology, University of Milano-Bicocca, 20126 Milan, Italy; 2 PhD Program in Neuroscience, Department of Medicine & Surgery, University of Milano-Bicocca, 20900 Monza, Italy; 3 Department of Neurorehabilitation Sciences, Casa di Cura Igea, 20144 Milan, Italy; 4 Laboratory of Neuropsychology, IRCCS Istituto Auxologico Italiano, 20122 Milan, Italy

**Keywords:** motor resonance, empathy, social cognition, transcranial magnetic stimulation, mirror neuron system, motor-evoked potentials

## Abstract

**Background/Objectives**: Empathy is essential for successful social functioning, mediating different aspects of social cognition in everyday life. An intriguing aspect is the involvement of empathy even in basic neural mechanisms of action perception, thanks to its association with the Mirror Neuron System (MNS). The present retrospective study explores whether individual differences in cognitive and affective empathy, measured by the Interpersonal Reactivity Index (IRI) questionnaire, can predict motor resonance—the enhancement of motor cortex reactivity during the observation of biological movements—during transitive and intransitive action observation. **Methods:** Data from 160 healthy subjects who participated in transcranial magnetic stimulation (TMS) experiments assessing corticospinal excitability during action observation were retrospectively analyzed using multiple linear regression models. Participants filled the IRI and observed intransitive single-digit finger movements (*n* = 80) or grasping actions directed at different targets (intransitive, object-directed, social-directed; *n* = 80) synchronized with TMS over the primary motor cortex, allowing the investigation of how action features modulate the relationship between participants’ empathic traits and motor resonance magnitude. **Results:** Results show that empathic traits do not affect motor resonance during intransitive movements, whereas they do when motor resonance is measured during the observation of transitive actions. Cognitive empathy, particularly the perspective-taking scale, significantly predicts motor resonance magnitude when observing goal-directed actions. Meanwhile, affective empathy, specifically the empathic concern scale, predicts motor resonance while observing social action. **Conclusions:** These findings highlight that different facets of empathy are significantly related to humans’ ability to understand others’ actions through inner simulation mechanisms, particularly concerning action goals and social relevance.

## 1. Introduction

Empathy is a multidimensional phenomenon that allows us to perceive, understand, and share another person’s affective state [1]. It plays a crucial role in social functioning, supporting social understanding, the formation and maintenance of interpersonal relationships, and prosocial behaviors [2,3]. Empathy is widely regarded as a construct involving cognitive and affective components [4,5,6]. The cognitive component refers to the ability to understand others’ mental states and emotions, partially overlapping with the Theory of Mind construct [7], while the affective component refers to sharing or resonating with others’ emotional experiences.

Considering the neural basis of empathy, a vast cerebral network is implied in its different facets, comprising the dorsolateral prefrontal cortex, the superior temporal cortex, the inferior parietal lobule, the insula, the anterior cingulate cortex, the precuneus, and subcortical structures such as the amygdala [8,9,10,11,12,13,14]. Cognitive empathy has been associated with the activity of the temporoparietal junction, medial prefrontal cortex, and the temporal pole. Affective empathy, on the other hand, engages the inferior frontal gyrus, anterior insula, cingulate cortices, the amygdala, and the medial orbitofrontal cortex [6,15,16,17,18,19,20].

Notably, several of these areas are also core regions of the Mirror Neuron System [MNS]—ref. [21], suggesting a link between this system and empathy. The hypothesis that mirroring mechanisms allow humans to internally simulate the emotions, actions, and sensations of others has prompted various theoretical accounts emphasizing their role in interpersonal relationships, where empathy is fundamental (e.g., [22,23,24,25]). However, the extent to which empathy contributes to mirroring mechanisms supporting action and understanding others’ intentions is still being debated (e.g., [26,27,28,29,30,31,32,33]).

Experimental evidence linking empathic skills with neurophysiological indexes of MNS activity is discordant and inconclusive. For instance, a recent meta-analysis concludes that cognitive and affective empathy are only moderately correlated with MNS activation [29]. A major source of this variability lies in the methodological heterogeneity across studies. Differences in empathy assessment approaches (e.g., self-report questionnaires vs. behavioral tasks, or emphasis on cognitive vs. affective components) and the use of diverse neurophysiological techniques (e.g., functional magnetic resonance imaging, electroencephalography, or transcranial magnetic stimulation—TMS) likely capture distinct dimensions of both MNS functioning and empathic processes. Consequently, these methodological disparities hinder the comparability of findings across studies, contributing to the fragmented and often ambiguous evidence regarding the link between mirror mechanisms and empathy [29].

In the present study, to better elucidate this relationship, we have focused on the predictive role of cognitive and affective empathy on a covert marker of MNS activation: the motor resonance phenomenon. Motor resonance is a basic action-perception matching mechanism that facilitates motor programs, mediating action recognition and understanding through the inner simulation of others’ actions [32]. According to MNS literature [34], it consists of the somatotopic enhancement of corticospinal excitability (CSE) assessed by delivering TMS over the primary motor cortex (M1) contingently upon action observation. Namely, motor-evoked potentials (MEPs) during the view of biological movements present higher amplitude than those recorded while observing a static body part; this effect is detectable only in the muscles involved in the observed movement [35,36,37]. The enhanced CSE during action observation is thought to reflect excitatory connections from the ventral premotor area, a key hub of the MNS; hence, it is considered a reliable proxy of MNS activation [38].

So far, few TMS studies have investigated the relationship between motor resonance magnitude and empathic dimensions during action observation [39,40,41,42,43,44]. All these studies measured participants’ empathy with self-report questionnaires (i.e., the empathy quotient—EQ [45]—or the interpersonal reactivity index—IRI [46]), correlating their scores with motor resonance indexes. In two works, Lepage and colleagues (2010, 2014) found that MEP facilitation during intransitive action observation was significantly related to the EQ total score [43,44]. Nevertheless, this correlation was not replicated in studies with a larger sample size or slightly different experimental designs [39,40]. Jola and coworkers (2012) found that scoring at the fantasy (FS) subscale of the IRI, measuring a facet of cognitive empathy, was positively related to MEP enhancement during the observation of Indian dance [41]. Finally, our research group found that motor resonance magnitude during the observation of grasping stimuli with social relevance was positively associated with the IRI’s empathic concern (EC) subscale, which measures affective empathy [42]. Overall, the available results suggest that empathy could influence motor resonance, but the specific contribution of its cognitive and affective components is still unclear due to inconsistent findings among studies. Thus, a more systematic investigation is required to draw firmer conclusions.

To this aim, in the present work, we retrospectively analyzed cognitive and affective facets of empathy, as assessed with the IRI questionnaire, from 160 healthy subjects who participated in a series of action observation experiments conducted by our research group in the last few years [42,47,48,49]. In these datasets, motor resonance was assessed by observing (a) intransitive, purposeless single-digit abductions or (b) whole-hand grasping movements that could be intransitive (i.e., the movement is presented in isolation, without any target), object-directed (i.e., a hand grasping a bottle), or social-directed (i.e., a hand grasping another hand—ref. [42]). With these action stimuli with different meanings and goals, we could investigate whether and how different dimensions of empathy may influence motor resonance.

We hypothesize that observers’ empathic traits could influence the magnitude of motor resonance during transitive and intransitive action observation with different engagement of empathy’s affective and cognitive dimensions, likely thanks to top–down influences mediated by high-order mental processing related to action understanding (e.g., [50,51,52,53]). In particular, affective empathy, involving sharing emotional experiences and affective states, should be more relevant in coding motor representations of socially relevant actions, as our socially directed grasping (e.g., [42]), which would likely lead to greater motor resonance responses according to the individual level of this empathic facet. In contrast, cognitive empathy, namely the ability to take the mental perspective of others, could be more critical for understanding an action goal, enabling one to make predictions about the outcome of a meaningful action (e.g., [4,20]). If this is true, we could expect that motor resonance responses during the observation of our object-directed stimulus are enhanced in individuals with higher cognitive empathy or perspective-taking abilities. Lastly, considering the possible contribution of distinct empathic dimensions for motor resonance magnitude during the observation of intransitive actions, we do not have a specific a priori hypothesis, given the controversial findings in the literature [39,40,43,44].

## 2. Materials and Methods

### 2.1. Participants

MEP and IRI data were taken from a series of published and unpublished studies on action observation conducted by our research group in the last 5 years [42,47,48,49] (Supplemental Table S1). Except for [42], none of these studies have explored the relation between motor resonance and participants’ empathic dimensions. These datasets included a total of 175 participants. Given the present work’s aim, we a priori excluded participants who were not native Italian speakers or did not fill out the IRI at the end of the experiment (*n* = 8). If the same participant took part in more than one experiment, we considered data only from the first experiment they took part in (*n* = 7). In the case of studies comprising multiple sessions or pre-post evaluations, we considered MEP data recorded during the baseline assessment of the first experimental session.

Following such criteria, data from 160 right-handed healthy Caucasian participants were considered. Half of them (35 males, mean age ± standard deviation − SD: 23.6 ± 2.9 years; mean education ± SD: 15.6 ± 2 years; mean Edinburgh Handedness Inventory score ± SD: 77 ± 14.1%) took part in experiments where motor resonance was recorded during the observation of intransitive single-digit movements (i.e., abduction of the index finger). The other half (34 males, mean age ± SD: 22.7 ± 3.2 years; mean education ± SD: 14.9 ± 2.4 years; mean Edinburgh Handedness Inventory score ± SD: 82.8 ± 14.7%) took part in experiments where motor resonance was recorded during the observation of whole-hand grasping movements that could be intransitive, object-, or social-directed. In both databases, motor resonance was always assessed from the contralateral hemisphere with respect to the hand performing the action presented in the visual stimulus. See Supplemental Table S1 for a detailed description of our final sample composition according to the datasets aggregated.

To assess whether this sample size per condition was sufficient to obtain reliable results from multiple linear regression models with four predictors (i.e., as the IRI subscales, our main target analysis, see Section 2.5), we ran an a priori power analysis with the software G*Power 3.1 [54]. The power analysis [*f*^2^ = 0.15—corresponding to a medium desired effect size [55], alpha error level: *p* = 0.05; statistical power = 0.9] suggested at least 73 participants to achieve enough statistical power.

All experimental procedures complied with the ethical standards outlined in the Declaration of Helsinki and were approved by the Ethics Committee of the University of Milano-Bicocca. Written informed consent was obtained from all participants before their inclusion in the study. No part of the study procedures or analysis plans were preregistered before the research was conducted. We report how we determined our sample size, all data exclusions, all inclusion/exclusion criteria, whether inclusion/exclusion criteria were established before data analysis, all manipulations, and all measures in the study. Stimuli, datasets, and analyses of this study are publicly available on Open Science Framework (OSF: https://osf.io/he8v9/ (accessed on 18 October 2025)).

### 2.2. Interpersonal Reactivity Index Questionnaire (IRI)

The IRI [46] is a self-report questionnaire that comprises the following four subscales of 7 items each: (a) Perspective Taking (PT, i.e., tendency to adopt the psychological point of view of others), (b) Fantasy (FS, i.e., tendency to transpose oneself imaginatively into the feelings of fictitious characters), (c) Empathic Concern (EC, i.e., ‘other-oriented’ feelings of sympathy and concern for others), and (d) Personal Distress (PD, i.e., ‘self-focused’ feelings of personal anxiety and unease in tense interpersonal setting). PT and FS subscales measure cognitive empathy, whereas EC and PD measure affective empathy. Higher scores indicate a higher level of empathy. Participants report the extent to which each of the 28 statements describes themselves on a 5-point Likert scale ranging from 0 (‘Does not ‘describe me well’) to 4 (‘Describes me very well’). IRI compilation takes about 10 min. In all the datasets, the Italian version of the IRI [56] was administered at the end of the final session of the experiment before debriefing participants on the aims of the specific experiment.

### 2.3. CSE Assessment During Action Observation

For all our datasets, CSE during action observation was assessed during passive action observation tasks. The paradigms were frame-based, consisting of the rapid presentation of two images: one showing the hand at rest from an egocentric perspective, and the other showing it in motion. The quick alternation between the two created the illusion of apparent movement. We instructed participants to observe these visual stimuli on a PC monitor while we delivered time-locked single-pulse TMS over M1. Participants’ hands were positioned out of view during the whole task. All action observation tasks were developed and run on the E-Prime software (E-Prime 2.0, Psychology Software Tools, Inc., Sharpsburg, PA, USA).

**Figure 1:**
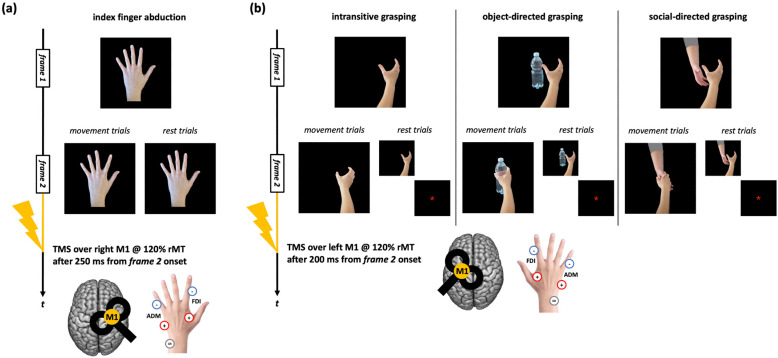
Action observation tasks used in the datasets considered for the present work. (**a**) Trials of the action observation tasks depicting index finger abduction movements. (**b**) Trials of the action observation tasks depicting right-hand grasping movements (intransitive, object-directed, or social-directed). The timing of the frames slightly varied according to the study from which MEP data were taken [for further information, see: refs. [42,47,48,49]]. ‘+’, ‘-’, and ‘GR’ indicate the positions of the positive, negative, and ground electrodes for FDI and ADM muscles EMG recording.

Given the aim of the present work, we considered only conditions that should induce a reliable motor resonance effect, i.e., where the action observed was performed with the upper limb contralateral to the TMS side and where TMS timing of administration was the optimal one for detecting CSE enhancement. For CSE recorded during the observation of index finger movements, the original tasks comprised blocks where participants observed static and moving abduction movements of the left-hand index finger [47,48,49]. During ‘action trials’, TMS was delivered over the right M1 after 250 ms from the onset of the movement frame to record corticospinal facilitation during action observation. During ‘rest trials’, TMS was delivered during the frame depicting the static hand to record baseline CSE (Figure 1a). For CSE assessed during the observation of grasping movements, the tasks comprised a series of blocks where participants observed right hands making whole-hand grasping, which could be intransitive, directed to a bottle, or directed to another hand [42]. During ‘action trials’, TMS was delivered over the left M1 after 200 ms from the onset of the movement frame. During ‘rest trials’, TMS was delivered while observing the frame depicting the static hand or an asterisk acting as fixation cross (Figure 1b).

### 2.4. TMS and EMG Recording

TMS pulses were delivered with a biphasic figure-of-eight coil (diameter = 70 mm) connected to a Magstim Rapid 2 (Magstim, Whitland, UK) or a Nexstim Eximia stimulator (Nexstim, Helsinki, Finland—see Supplemental Table S1).

At the start of each session, the motor hotspot of the first dorsal interosseous (FDI) muscle was identified by systematically moving the coil in 5 mm increments around the presumed hand motor area while delivering slightly suprathreshold stimuli and recording MEPs. For action observation tasks where participants observed the index finger’s abduction movements, the right hemisphere’s FDI hotspot was targeted; for tasks where grasping movements were presented, the left hotspot was stimulated. According to the specific experiment [see [42,47,48,49] and Supplemental Table S1], the individual rMT was calculated as the minimum TMS intensity (expressed as the percentage of maximum stimulator output) able to elicit an MEP of at least 50 µV in FDI 5 times out of 10 [57] or through a parameter estimation by sequential testing (PEST) procedure [58]. TMS intensity was set at 120% rMT during all the action observation tasks. Throughout the experimental sessions, TMS coil position and stability were continuously monitored using neuronavigation systems. Specifically, SofTaxic Optic 3 (EMS, Bologna, Italy) was employed for data acquired with the Magstim Rapid 2 stimulator, whereas the Nexstim navigated brain stimulation system (Nexstim, Helsinki, Finland) was used for recordings obtained with the Nexstim Eximia stimulator. The coil was always placed tangentially to the scalp and tilted 45° to the midline (positioned perpendicular to the stimulated cortical gyrus), inducing currents in the brain with an anterior-to-posterior (first phase)/posterior-to-anterior (second phase) direction.

MEPs during action observation tasks were recorded from the FDI and the abductor digiti minimi (ADM) muscles of the hand contralateral to the TMS site. We placed active electrodes over the muscle bellies and reference ones over the metacarpophalangeal joint of the index (for FDI) and little finger (ADM). The ground electrode was placed over the ipsilateral head of the ulna. We recorded MEPs with Signal software (Cambridge Electronic Devices, Cambridge, UK) at a sampling rate of 5000 Hz through a Digitimer D360 amplifier (Digitimer Ltd., Welwyn Garden City, UK) connected to a CED micro1401 A/D converter (Cambridge Electronic Devices, Cambridge, UK). The signal was amplified during data acquisition, band-pass (10–1000 Hz), and notch filtered. Single-trial EMG data were collected in a time window of 300 ms, i.e., from 100 ms before to 200 ms after the TMS pulse. We analyzed MEPs offline using Signal software (version 3.13). Given that MEP extraction parameters (i.e., time window considered for MEP peak-to-peak and cut-offs for artifactual trials labeling) slightly varied across the preprocessing of our original studies, we decided to keep extraction parameters constant in the present work, re-analyzing MEPs for datasets where they changed from established criteria [i.e., [47,48]]. So, for all our datasets, MEP peak-to-peak amplitude was calculated in each trial between 5 ms and 60 ms from the TMS pulse. We excluded from the analysis trials with artifacts deviating from 100 µV in the 100 ms before the TMS pulse and trials in which MEP amplitude was smaller than 50 µV [as in [42,49]]. On average, adopting these criteria for each participant, 3.9 ± 3.1% of recorded trials were excluded.

### 2.5. Statistical Analyses

We computed a *motor resonance index* [59] to assess corticospinal facilitation during action observation as the ratio in MEP amplitude between movement and rest trials:

$$motor\;resonance\;index\;(\%)=\frac{MEP\;amplitude\;in\;movement\;trials}{MEP\;amplitude\;in\;rest\;trials}\;-1$$

Namely, for every participant and condition (i.e., left-hand index finger abduction, intransitive grasping, object-directed grasping, and social-directed grasping), the mean MEP amplitude recorded during movement trials was normalized by dividing it by the MEP amplitude from rest trials of the same condition, which served as the baseline measure of CSE. To express modulation relative to rest, the value ‘1’ was subtracted from this ratio. Consequently, positive values reflected CSE facilitation during action observation, indicating the presence of motor resonance. Raw MEP values in each condition and muscle are reported in Supplemental Tables S2 and S3.

**Figure 2:**
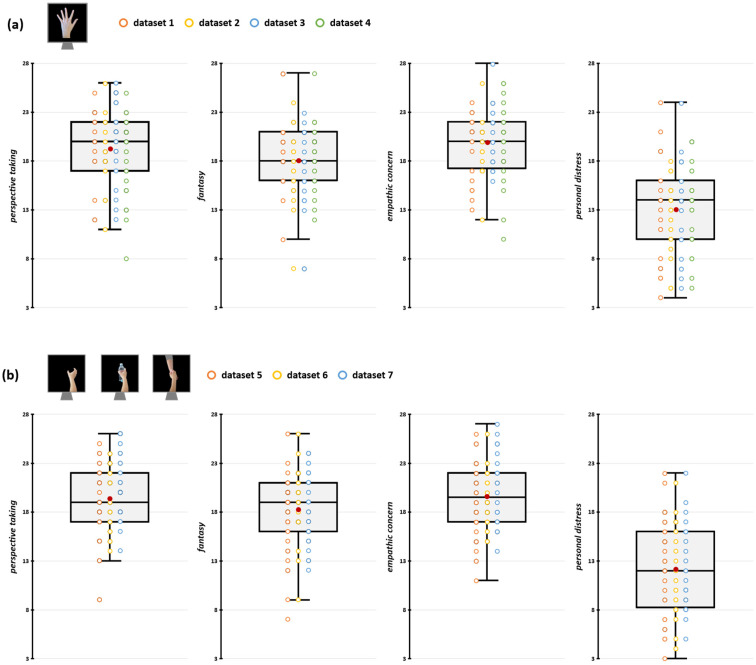
Participants’ IRI scores for datasets depicting index finger abduction (**a**) and grasping movements (**b**). In the box-and-whiskers plots, red dots indicate the means of the distributions. The center line denotes their median values. Colored dots show individual participants’ scores according to the specific dataset from which data were collected. The box contains the 25th to 75th percentiles of the dataset. Whiskers extend to the largest observation falling within the 1.5× inter-quartile range from the first/third quartile.

We preliminarily conducted a series of sanity checks to assess whether methodological differences between the aggregated datasets have impacted their comparability. Firstly, we assessed whether differences in TMS stimulators and rMT determination procedures (see Section 2.4) may have influenced MEP amplitude recorded during the action observation tasks. To this aim, we conducted two repeated measures analyses of variance (rmANOVA) on raw MEP amplitudes during rest trials with the within-subjects factor ‘Muscle’ (FDI, ADM) and the between-subjects factor ‘Stimulator’ (Magstim, Nexstim) or ‘rMT determination procedure’ (PEST, 5/10). Results showed no statistically significant effects of either between-subjects factors or of their interaction with the factor ‘Muscle’ (all *F*s < 1.51; all *p*s > 0.221; see Supplemental Tables S4 and S5), suggesting that differences in TMS settings across experiments did not influence corticospinal excitability magnitude. Then, we assessed whether the IRI scores in the samples of the datasets we aggregated were comparable (i.e., none of our original experiments presented samples having empathic scores that are significantly lower/higher than the others) by running four one-way ANOVA (one for each subscale of the IRI) with the 7-level factor ‘Dataset’. We found no significant differences in empathic dimensions among the seven experiments’ samples (all *F*s < 1.55, all *p*s > 0.166; see Supplemental Table S6). IRI scores of the participants in the different datasets are depicted in Figure 2 and Table 1.

As a further positive control, we checked whether muscle-specific motor resonance can be detected while observing our movement stimuli. To this aim, we conducted repeated-measures ANOVAs on *motor resonance index* values with within-subject factor ‘Muscle’ (FDI, ADM)—for data recorded during the observation of index finger abduction—and factors ‘Grasping type’ (intransitive, object-directed, social-directed) and ‘Muscle’ (FDI, ADM)—for data collected during the observation of grasping movements. In both analyses, we also introduced the between-subjects factor ‘Dataset’ (with 4 levels for the rmANOVA on data recorded during the observation of index finger abduction and 3 levels for data recorded during the observation of grasping movements) to check whether motor resonance patterns were similar among the works that we aggregated.

Lastly, looking for possible relations between motor resonance patterns and participants’ emphatic dimensions, for every type of movement observed, we run multiple linear regression models with FDI corticospinal facilitation effects during action observation (i.e., *motor resonance index*) as the dependent variable and the four IRI subscales (i.e., PT, FS, EC, PD) as predictors. The same models were also run with the two aggregated scales measuring cognitive and affective empathy constructs (given by the sum of PT and FS; EC and PD scores, respectively) as predictors [for a similar procedure see, e.g., [33]]. To take into account the possible effects of participants’ sex, which is known to influence empathic abilities [e.g., [60,61]], we have also considered the between-subjects factor ‘Sex’ (Male, Female) in these models.

All statistical analyses were performed using the software Jamovi [v. 2.6.24; [62]]. Multiple linear regression models were run with the Jamovi package GAMLj [63]. Statistical significance was set at *p* < 0.05. Normality of *motor resonance index* and IRI scores distributions was confirmed for all our variables, checking it with Q-Q plots assessment and skewness/kurtosis values [which must be between −2/2—[64]]. For rmANOVAs, data sphericity was tested by applying Mauchly’s test in every dataset. When data sphericity was not confirmed, the Greenhouse–Geisser correction was applied. Significant effects were further explored with multiple Bonferroni-corrected post hoc comparisons, if not otherwise specified. For all our multiple linear regression models, homogeneity of the residual variance and normality of the residuals were confirmed through Breusch–Pagan and Kolmogorov–Smirnov tests, respectively (all *p*s > 0.058). Partial eta-squared (η_p_^2^), Cohen’s *d*, and adjusted coefficient of determination (R^2^_adj_) were calculated in every rmANOVA, *t*-test, and multiple regression, respectively, and reported as effect size values. We also reported the standardized regression coefficient (β) for multiple regressions. Finally, in case of null findings from multiple linear regression models, to better estimate the evidence in favor of the null hypothesis, we have also conducted the respective Bayesian version to determine the Bayes Factor (i.e., BF_01_) using the Jamovi package ‘jsq’ [65]. In the Section 3, mean ± SE is reported for each variable.

## 3. Results

### 3.1. Muscle-Specific CSE Enhancement During Action Observation

**Figure 3:**
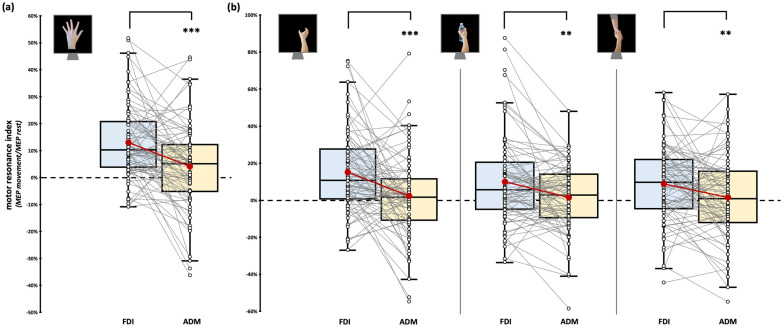
*Motor resonance index* for FDI (blue boxplot) and ADM (yellow boxplot) muscles. (**a**) *Motor resonance index* values recorded during the observation of intransitive index finger abduction movements. (**b**) *Motor resonance index* values recorded during the observation of intransitive, object-related, and social-related grasping movements. In the box-and-whiskers plots, red dots indicate the means of the distributions. The center line denotes their median values. White dots show individual participants’ scores. The box contains the 25th to 75th percentiles of the dataset. Whiskers extend to the largest observation falling within the 1.5× inter-quartile range from the first/third quartile. Significant *p* values of post hoc comparisons are reported (** = *p* < 0.01; *** = *p* < 0.001).

Considering the observation of intransitive index finger abduction movement, we found significant effect of factor ‘Muscle’ (*F*_1,76_ = 7.64, *p* = 0.007, η_p_^2^ = 0.09). FDI *motor resonance index* during the observation of index finger abduction movements (12.96 ± 1.54%) is significantly greater than in ADM (4.14 ± 1.79%, *t*_76_ = 4.34, *p_Bonf_* < 0.001, *d* = 0.49; Figure 3a). Crucially, the rmANOVA showed no statistically significant effect of the ‘Muscle’ X ‘Dataset’ interaction (*F*_3,76_ = 0.16, *p* = 0.922, η_p_^2^ < 0.01), suggesting that muscle-specific motor resonance is detectable in all the datasets we aggregated.

Considering the observation of grasping movement, we only found a significant effect of the factor ‘Muscle’ (*F*_1,77_ = 21.59, *p* < 0.001, η_p_^2^ = 0.22). Namely, regardless of the grasping observed, motor resonance values found from FDI were always greater than ADM ones. As expected, stimuli-specific planned comparisons confirmed that all our grasping stimuli led to muscle-specific CSE facilitation during action observation (intransitive grasping: FDI motor resonance: 15.05 ± 2.47% vs. ADM motor resonance: 2.59 ± 2.56%, *t*_77_ = 4.01, *p* < 0.001, *d* = 0.45; object grasping: FDI motor resonance: 9.83 ± 2.66% vs. ADM motor resonance: 1.32 ± 2.04%, *t*_79_ = 3.4, *p* = 0.001, *d* = 0.38; social grasping: FDI motor resonance: 8.88 ± 2.32% vs. ADM motor resonance: 1.51 ± 2.47%, *t*_79_ = 2.69, *p* = 0.009, *d* = 0.3; Figure 3b). Similarly to the previous analysis, the rmANOVA showed no statistically significant effects of the between-subject factor ‘Dataset’ (all *F*s < 3.19, all *p*s > 0.07), suggesting that the patterns previously described are detectable regardless of the specific experiment considered. ‘Grasping type’ (*F*_2,154_ = 1.61, *p* = 0.203, η_p_^2^ = 0.02) and ‘Grasping Type’ X ‘Muscle’ interaction (*F*_1.8,134.2_ = 1.49, *p* = 0.231, η_p_^2^ = 0.02) did not reach statistical significance.

Given that we found muscle-specific motor resonance patterns in all our analyses and for all our visual stimuli, we ran regression models on IRI scores only for FDI *motor resonance index* values. For the sake of completeness, the same regression models were also run for the ADM *motor resonance index,* showing no statistically significant effect in any of our conditions, suggesting that IRI scores–motor resonance magnitude relations are muscle-specific (see Supplemental Tables S7 and S8, Supplemental Figures S1 and S2). These results also suggest that the minor variations in task parameters (see Section 2.3) are unlikely to have affected motor resonance magnitude.

### 3.2. Empathic Modulation of Motor Resonance for Index Finger Abduction Movements

**Figure 4:**
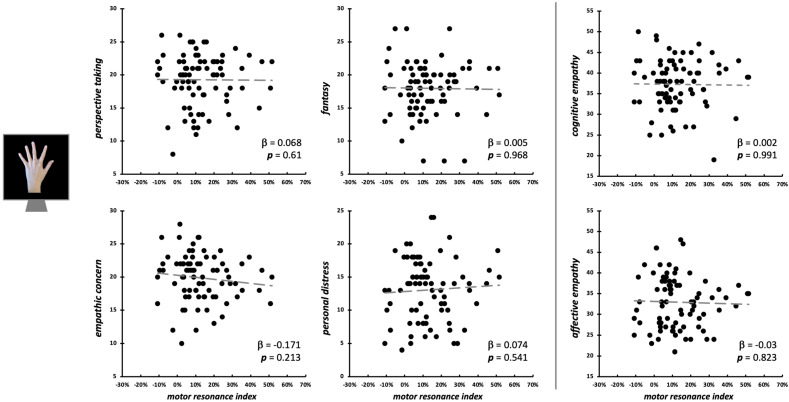
Scatterplot between (FDI) *motor resonance index* during intransitive index finger abduction movements observation and IRI scores (**left panels**: four subscales—PT, FS, EC, PD; **right panels**: cognitive and affective empathy aggregated scales). Standardized multiple regression coefficients and *p*-values are reported in the lower corner of the scatterplot. Gray lines indicate linear regression lines.

Considering the observation of intransitive index finger movements, neither the four IRI subscales (*F*_4,75_ = 0.46, *p* = 0.758, R^2^_adj_ < 0.01), nor cognitive and affective empathy aggregated scales (*F*_2,77_ = 0.03, *p* = 0.969, R^2^_adj_ < 0.01) significantly predicted *motor resonance index* values (Figure 4). In both regression models, no differences intercurred between males and females when the between-subjects factor ‘Sex’ is considered (all *F*s < 1.42, all *p*s > 0.237). Considering the null findings, we also ran Bayesian linear regressions to quantify the evidence in favor of the null hypothesis (i.e., empathic dimensions did not predict motor resonance magnitude). Here, for both the model with the four IRI subscales (PT: BF_01_ = 4.3; EC: BF_01_ = 3.1; FS: BF_01_ = 4.3; PD: BF_01_ = 3.9;) and the one with the aggregated scales (cognitive empathy: BF_01_ = 4.3; affective empathy: BF_01_ = 4.2), evidence in favor of the null hypothesis is moderate (i.e., BF_01_ > 3) [66].

### 3.3. Empathic Modulation of Motor Resonance for Grasping Movements

**Figure 5:**
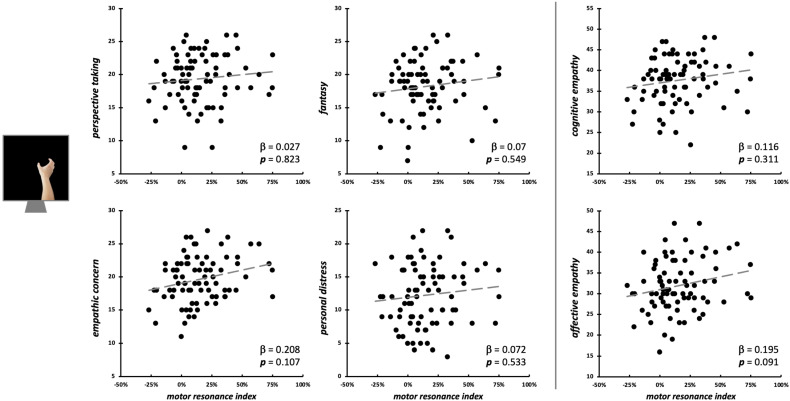
Scatterplot between (FDI) *motor resonance index* during intransitive grasping observation and IRI scores (**left panels**: four subscales—PT, FS, EC, PD; **right panels**: cognitive and affective empathy aggregated scales). Standardized multiple regression coefficients and *p*-values are reported in the lower corner of the scatterplot. Gray lines indicate linear regression lines.

During the observation of intransitive grasping movements performed with the whole hand, motor resonance was not predicted by either the four IRI subscales (*F*_4,75_ = 1.49, *p* = 0.215, R^2^_adj_ = 0.02), nor cognitive/affective empathy values (*F*_2,77_ = 2.59, *p* = 0.082, R^2^_adj_ = 0.04) (Figure 5). As for index finger abductions, we run Bayesian linear regression models to quantify the evidence in favor of the null hypothesis [66]. Here, for PT (BF_01_ = 2.96), FS (BF_01_ = 2.21), PD (BF_01_ = 2.67), and cognitive empathy (BF_01_ = 1.68), evidence in favor of the null hypothesis is weak (i.e., 0 < BF_01_ < 3). For EC (BF_01_ = 0.45, corresponding to BF_10_ = 2.22) and affective empathy (BF_01_ = 0.73, corresponding to BF_10_ = 1.37), there is instead weak evidence (1 < BF_10_ < 3) for the alternative hypothesis (i.e., that these two empathic dimensions could influence motor resonance).

**Figure 6:**
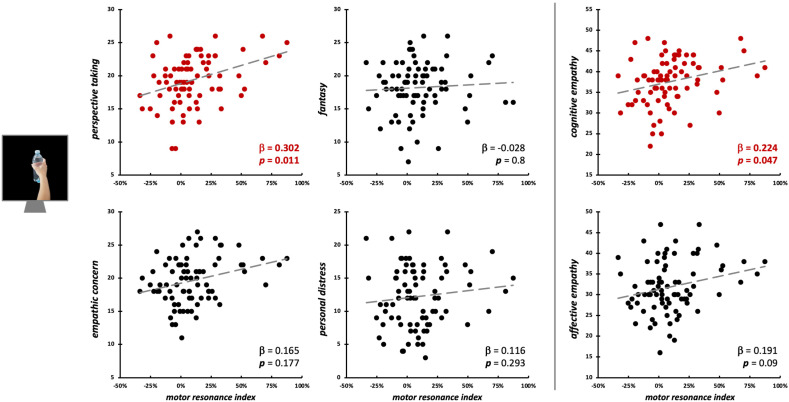
Scatterplot between (FDI) *motor resonance index* during object-directed grasping observation and IRI scores (**left panels**: four subscales—PT, FS, EC, PD; **right panels**: cognitive and affective empathy aggregated scales). Standardized multiple regression coefficients and *p*-values are reported in the lower corner of the scatterplot. Gray lines indicate linear regression lines. In red: significant relations.

Motor resonance during the observation of object-directed grasping was significantly predicted only by PT scores (β = 0.3, *t*_75_ = 2.62, *p* = 0.011; model fit: *F*_4,75_ = 3.72, *p* = 0.008, R^2^_adj_ = 0.12) and by cognitive empathy (β = 0.22, *t*_77_ = 2.02, *p* = 0.047; model fit: *F*_2,77_ = 4.67, *p* = 0.012, R^2^_adj_ = 0.09) (Figure 6; Table 2).

**Figure 7:**
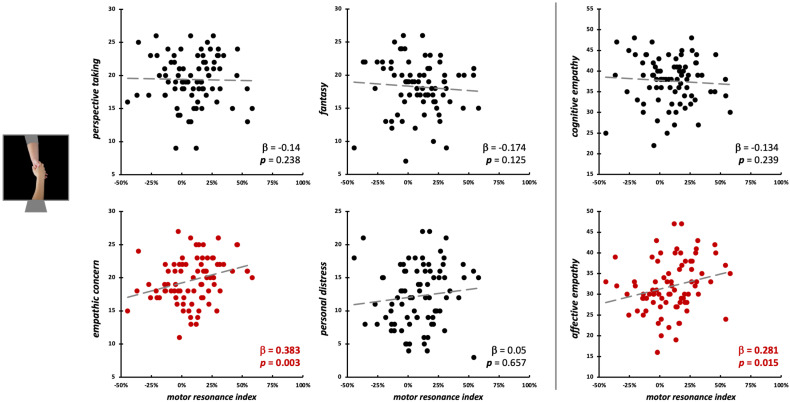
Scatterplot between (FDI) *motor resonance index* during social-directed grasping observation and IRI scores (**left panels**: four subscales—PT, FS, EC, PD; **right panels**: cognitive and affective empathy aggregated scales). Standardized multiple regression coefficients and *p*-values are reported in the lower corner of the scatterplot. Gray lines indicate linear regression lines. In red: significant relations.

Conversely, during the observation of social-directed grasping, we found a sort of opposite pattern: here, EC scores (β = 0.38, *t*_75_ = 3.11, *p* = 0.003; model fit: *F*_4,75_ = 2.88, *p* = 0.028, R^2^_adj_ = 0.09) and affective empathy (β = 0.28, *t*_77_ = 2.48, *p* = 0.015; model fit: *F*_2,77_ = 3.25, *p* = 0.044, R^2^_adj_ = 0.05) significantly predicted the *motor resonance index* values (Figure 7; Table 2).

Similarly, for intransitive index finger abduction movements observation, the between-subjects factor ‘Sex’ did not affect the associations between IRI scores and motor resonance for grasping movements (all *F*s < 0.93, all *p*s > 0.342).

## 4. Discussion

The present study shows that the degree of participants’ self-reported empathy predicts the magnitude of a covert marker of MNS activation in humans: the motor resonance phenomenon [i.e., CSE enhancement during biological movements observation, [34]]. However, and this is the most novel and interesting result, different facets of empathy are associated with motor resonance depending on the type of action we are observing. On the one hand, only during the observation of movements that allude to an intention (i.e., transitive actions), motor resonance is modulated by empathy; on the other, the action’s purpose and the presence of interpersonal contact with another agent determine whether empathy’s cognitive or affective component influences the phenomenon’s magnitude.

The key finding is that participants’ empathic traits predict the degree of motor resonance elicited by observing both object- and social-directed movements, but with a sort of ‘double dissociation’ in their pattern. Namely, motor resonance magnitude during the observation of object-directed grasping actions is predicted by participants’ cognitive empathy, particularly by perspective-taking scores (PT subscale; i.e., the tendency to adopt the other’s psychological point of view). Conversely, affective empathy, especially the empathic concern component (EC subscale; i.e., the capacity to feel and respond with sympathy to others’ emotions), predicts motor resonance selectively during the view of grasping actions, implying interpersonal bodily contact. This evidence underlines that the relation between this covert marker of MNS activation and empathic dimensions is not ‘all-or-nothing’ but is influenced by many features of the observed action, like its goal, meaning, and social value.

With respect to cognitive empathy, the present result confirms previous evidence of the existence of a relationship between perspective-taking abilities and MNS activation during the observation of both simple and complex transitive actions [e.g., [28,29,67,68,69,70,71]]. In our study, participants with a stronger tendency to adopt others’ perspectives appeared to be facilitated in sharing the motor representation of object-directed actions. We speculate that this effect may reflect facilitation to internally represent the target of the observed action, thereby supporting the attribution of intentionality. This facilitation can be underpinned by the greater influence of MNS over premotor areas [38], leading, in turn, to a greater magnitude of the motor resonance phenomenon.

Affective empathy was called into question in social interactions and emotion processing. Both visuo-motor and visuo-tactile mirroring mechanisms are related to EC, especially when the observed actions are featured by sensational, emotional, and affective meanings [30,42,72,73,74,75,76,77,78,79]. Our findings confirmed this relationship, revealing that participants with a higher level of affective empathy exhibited stronger motor resonance when observing an action implying interpersonal contact, such as a hand grasping another hand. Likely, this association may reflect a deeper embodied simulation of the observed social gesture, allowing observers to better resonate with others’ emotions and sensations, favoring the vicarious recruitment of motor areas driven by the MNS.

Notably, the dissociation of cognitive and affective empathy concerning their influence on motor resonance according to the action goal (i.e., object vs. human body parts) aligns with the different neural substrates of these processes. As already said in the Introduction, cognitive empathy has been associated with the activity of the temporoparietal junction, medial prefrontal cortex, and the temporal pole. In contrast, affective empathy engages the inferior frontal gyrus, anterior insula, cingulate cortices, the amygdala, and the medial orbitofrontal cortex [e.g., [6,15,16,17,18,19,20]]. MNS literature highlighted that the observation of object-directed movements activated temporoparietal regions to a greater extent, while observing actions with social or communicative features consistently recruits more frontal and limbic areas [e.g., [80,81,82,83,84]]. Altogether, this evidence suggests an overlap of the areas recruited by the MNS during specific action observation and those engaged in cognitive and affective empathy, corroborating the distinct regression patterns found in our data.

Another important finding of our study is that empathic traits do not predict motor resonance for intransitive movement observation, like single-finger abductions or whole-hand grasping (even if, in this latter case, Bayesian statistics show that EC and affective empathy scores could be weakly related to motor resonance magnitude). This finding is in line with previous TMS evidence [39,40], with only one study finding a correlation between the EQ questionnaire [45] and CSE enhancement during intransitive action observation, but in a very small sample size [*n* = 10, 43]. It is reasonable to expect that the observation of intransitive movements, being purposeless and not directed to a target or with a specific goal, activated the MNS (and hence premotor connections with M1) more automatically and feed-forwardly. This likely occurs due to the minimal need for additional cognitive or affective top–down processing of observed actions, which typically engages MNS regions involved in higher-order aspects of action understanding [e.g., vitality form, social affordance, movement intention—refs. [52,85,86,87]], including empathy [12,88]. We can speculate that only when the observed action is goal-directed and more cognitively demanding, e.g., presenting a target and then a meaning, the magnitude of motor resonance (and, in turn, MNS recruitment) starts to be influenced by the participant’s empathic dimensions, reflecting the involvement of a greater load of processing during action observation. In this context, observing a purposeless finger abduction movement is less cognitively demanding than observing an intransitive grasping movement [89], elucidating why, for this latter stimulus, Bayesian regression models suggest that there could be (weak) evidence for a possible relationship between affective empathy dimensions and motor resonance, which future studies could deepen.

Taken together, our results highlight that different empathic dimensions modulate MNS recruitment and corticospinal motor resonance according to the features of the observed actions. This could partially explain the conflicting evidence found in the literature exploring the MNS relationship with empathic dimensions, given that different tasks and visual stimuli of movement are usually adopted between and within studies [29]. From a broader perspective, our findings suggest that inner motor simulation during action observation can occur through two orders of processes [37,41]. One involves direct motor resonance, leading to a kinematic alignment between the observed and simulated intransitive, meaningless movements that do not need an empathic mediator. The other involves more complex and top-down processes, likely mediated by widespread cerebral networks, where cognitive and affective empathic processes can assist mirroring mechanisms recruited during the observation of transitive, goal-directed actions aimed at supporting interactions with objects or other people. As the complexity of the observed movement increases (e.g., from intransitive single-digit movements to transitive ones with a goal/target) or when a second agent is involved in the observed movement, these routes are complementarily recruited, and different facets of empathic processing influence MNS activation, leading to varying magnitudes of motor resonance phenomena. Overall, our findings corroborate the hypothesis that empathy is not merely a by-product of MNS activation and mirror activity but, instead, a multidimensional construct underpinned by interactions among a vast number of brain networks, comprising, but not limited to, the MNS [8,19,26,29].

In the future, systematic investigations like the present ones could be extended to other neurophysiological markers related to MNS recruitment during action observation (e.g., changes in blood-oxygenation level-dependent responses, mu-rhythm desynchronization). This would allow us to assess whether participants’ empathic features influence them with patterns similar to the ones found for the motor resonance phenomenon. Finally, the present results also have potential implications for motor learning [90], given that empathic abilities may influence motor skill acquisition [91,92].

As a last note, our retrospective study presents some methodological aspects that should be stressed.

Firstly, our results are based on a self-reported measure of empathy (i.e., the IRI), which captures participants’ perceived empathic tendencies. Self-reported measures are widely used to explore empathy dimensions, and the IRI is one of the most widely used instruments. However, this type of measure may be limited by bias in self-perception or social desirability effects [29,93]. Future studies could also investigate the motor resonance–empathy relationship with implicit behavioral or physiological measures of empathic dimensions, like implicit association tasks, skin conductance paradigms, or heartbeat rate-based measurements [e.g., [94,95,96,97]].

Secondly, although the sample size is overall adequate for the proposed analyses (see Section 2.1), significant regressions present small-to-medium effect size values—i.e., R^2^_adj_ are comprised between 0.05 and 0.12 and β values between 0.2 and 0.4 [98]. This evidence highlights that empathic dimensions explain just a small portion (i.e., from 5 to 12%) of motor resonance phenomenon variance, corroborating the evidence that the relation between MNS and empathy is low-to-moderate [29] and suggesting that large samples are required to detect these effects successfully.

Thirdly, action observation tasks from which we took motor resonance data present highly predictable stimuli (i.e., the same movement was always presented in a block design, see Section 2.3). This could have influenced MNS and empathic networks recruitment, influencing the magnitude of the relationship between CSE facilitation and individuals’ empathic dimensions. Indeed, when actions are highly predictable, observers may rely primarily on automatic simulation processes. In contrast, the observation of less predictable or ambiguous goal-directed actions might require greater engagement of inferential and perspective-taking mechanisms, e.g., to anticipate the actor’s intentions [99,100,101]. This increased cognitive demand could strengthen the association between perspective-taking abilities and neural activity within regions supporting action understanding, such as the inferior parietal and temporoparietal cortices [102], in turn leading to greater modulation of the motor resonance phenomenon according to an individual’s empathic predisposition. Future studies could systematically manipulate action predictability to clarify how variability in goal-directedness shapes the interplay between simulation-based and inferential components of empathy and action understanding.

Again, it could be argued that, during the observation of intransitive index-finger abduction, motor resonance was measured in the non-dominant motor cortex (i.e., right M1), whereas, during grasping observation, it was assessed in the dominant motor cortex (i.e., left M1). This methodological difference might represent a potential confound. However, it is worth noting that, at least when observing unilateral hand movements presented from an egocentric perspective, as in our datasets, previous studies have consistently reported that motor resonance exhibits a pattern of hemispheric lateralization, with its detection in the target motor system being strongest during the observation of movements performed by the contralateral hand [59,103,104]. According to this hemispheric mechanism of the motor resonance, CSE was assessed in all our tasks by stimulating the M1 contralateral to the hand that performed the observed movement. Hence, considering that, in both action observation tasks, we stimulated the optimal hemisphere for the features of the visual stimuli depicted, the absence of significant relationships between motor resonance and empathic dimensions during intransitive finger abduction conditions is not due to the stimulation of a different hemisphere with respect to grasping conditions. Nevertheless, to date, less is known about the possible hemispheric specificity of motor resonance–empathy relations, and future studies could also deepen this aspect.

Last but not least, given the nature of our experimental hypothesis, our work focused on the possible predictive role of empathic dimensions on MNS recruitment. Future studies could also explore how and if MNS functioning predicts participants’ empathic dimensions (i.e., the opposite direction to the one explored here).

## 5. Conclusions

In conclusion, our study shows that motor resonance magnitude, a non-invasive marker of MNS activity in humans, can be partially predicted by individuals’ empathic dimensions. This relation varies across distinct empathy domains according to the features and the complexity of the observed actions. Our findings suggest that cognitive and affective empathy can facilitate the magnitude of motor resonance and, in turn, MNS recruitment. Still, they are differentially implicated in mirroring mechanisms depending on the target of the action, its social role, and the actor’s intention. If the relationships found in the present retrospective investigation will be corroborated, our results could open up to the investigation of motor resonance impairments as a potential signature of psychiatric or neurodevelopmental disorders presenting impaired empathic skills [e.g., [88,105,106,107,108,109]]. Future research should investigate whether disorders affecting specific empathy components are also associated with action-specific alterations in motor resonance, and more broadly, with disruptions in MNS functioning.

## Figures and Tables

**Table 1:** IRI scores (mean ± ES) at the different subscales according to the single dataset sample. No significant differences in IRI scores occurred across the datasets aggregated in the present work (all *F*s < 1.55, all *p*s > 0.166; see Supplemental Table S3).

Dataset Number	PT	FS	EC	PD	Cognitive Empathy	Affective Empathy
1	19.67 ± 0.88	19.17 ± 0.94	19.06 ± 0.85	12.61 ± 1.35	38.83 ± 1.17	31.67 ± 1.63
2	18.75 ± 0.93	17.2 ± 1.02	19.5 ± 0.76	12.31 ± 1.01	35.94 ± 1.47	31.81 ± 1.28
3	20.23 ± 0.87	16.91 ± 0.95	20.55 ± 0.63	13.77 ± 1.06	37.14 ± 1.53	34.32 ± 1.47
4	18.5 ± 0.85	18.58 ± 0.68	20.13 ± 0.86	13.25 ± 0.79	37.08 ± 1.22	33.38 ± 1.22
5	18.3 ± 0.69	17.71 ± 0.65	19.1 ± 0.64	12.62 ± 0.81	36 ± 0.99	31.71 ± 1.16
6	19.46 ± 0.68	18.77 ± 0.91	19.5 ± 0.57	11.41 ± 1.04	38.23 ± 1.16	30.82 ± 1.05
7	20.83 ± 0.67	18.5 ± 0.76	20.67 ± 0.73	12.54 ± 0.91	39.33 ± 1.06	33.21 ± 1.39

**Table 2:** Results from statistically significant linear regression models on IRI subscales and aggregated scales for motor resonance recorded during transitive action observation. In bold: statistically significant predictors.

Stimulus	Model	Predictor	Estimate	SE	β	t	*p*
Object-directed grasping	IRI subscales	(Intercept)	0.098	0.025		3.942	<0.001
		**PT**	**0.019**	**0.007**	**0.302**	**2.623**	**0.011**
		FS	−0.002	0.007	−0.028	−0.255	0.8
		EC	0.011	0.008	0.165	1.361	0.177
		PD	0.006	0.006	0.116	1.058	0.293
	IRI aggregated scale	(Intercept)	0.098	0.025		3.863	<0.001
		**Cognitive empathy**	**0.009**	**0.005**	**0.224**	**2.017**	**0.047**
		Affective empathy	0.007	0.004	0.191	1.717	0.09
Social-directed grasping	IRI subscales	(Intercept)	0.089	0.022		4.004	<0.001
		PT	−0.008	0.007	−0.14	−1.189	0.238
		FS	−0.009	0.006	−0.174	−1.552	0.125
		**EC**	**0.023**	**0.007**	**0.383**	**3.106**	**0.003**
		PD	0.002	0.005	0.05	0.446	0.657
	IRI aggregated scale	(Intercept)	0.089	0.023		3.934	<0.001
		Cognitive empathy	−0.005	0.004	−0.134	−1.187	0.239
		**Affective empathy**	**0.009**	**0.004**	**0.281**	**2.482**	**0.015**

## Data Availability

The present study’s dataset, analyses, and stimuli are publicly available on Open Science Framework (OSF) at the following link: https://osf.io/he8v9 (accessed on 18 October 2025).

## Supplementary Materials

The following supporting information can be downloaded at: https://www.mdpi.com/article/10.3390/brainsci15111174/s1, Supplemental Table S1. Sample size composition in the seven datasets aggregated in the work. Supplemental Table S2. Mean ± standard error (SE) of the raw MEPs recorded from FDI in the different experimental conditions. Paired samples *t*-tests between rest and action trials are also reported. Supplemental Table S3. Mean ± standard error (SE) of the raw MEPs recorded from ADM in the different experimental conditions. Paired samples *t*-tests between rest and action trials are also reported. Supplemental Table S4. Results from the ‘Muscle’ (FDI, ADM) X ‘Stimulator model’ (Magstim, Nexstim) repeated measures ANOVA (rmANOVA) run on MEPs recorded during ‘rest’ trials to check whether the two stimulators adopted for MEP assessment in the aggregated datasets affect corticospinal excitability during action observation. Supplemental Table S5. Results from the ‘Muscle’ (FDI, ADM) X ‘rMT determination procedure’ (PEST, 5/10) repeated measures ANOVA (rmANOVA) run on MEPs recorded during ‘rest’ trials to check whether the two resting motor threshold procedures adopted in the aggregated datasets affect corticospinal excitability during action observation. Supplemental Table S6. Results from the one-way ANOVAs run to check whether the IRI scores in the samples of the studies we aggregated were comparable. A one-way ANOVA with the 7-level factor ‘Dataset’ was performed for each IRI subscale (PT, FS, EC, PD). Supplemental Table S7. Results from the multiple linear regression models run to check whether the IRI scores (i.e., the 4 subscales and 2 aggregated constructs) are predictive of ADM *motor resonance index* during the observation of intransitive index finger abduction movements. Supplemental Table S8. Results from the multiple linear regression models run to check whether the IRI scores (i.e., the 4 subscales and 2 aggregated constructs) are predictive of ADM *motor resonance index* during the observation of grasping movements. Supplemental Figure S1. Scatterplot between (ADM) *motor resonance index* for intransitive movements (a: index finger; b: grasping) and IRI scores (left panels: four subscales—PT, FS, EC, PD; right panels: cognitive and affective empathy constructs). Standardized multiple regression coefficients and *p*-values are reported. Supplemental Figure S2. Scatterplot between (ADM) *motor resonance index* for transitive grasping movements (a: object-directed grasping; b: social-directed grasping) and IRI scores (left panels: four subscales—PT, FS, EC, PD; right panels: cognitive and affective empathy constructs). Standardized multiple regression coefficients and *p*-values are reported.

## Abbreviations

The following abbreviations are used in this manuscript:

ADM	Abductor digiti minimi
CSE	Corticospinal excitability
EC	Empathy Concern
FDI	First dorsal interosseus
FS	Fantasy
IRI	Interpersonal Reactivity Index
M1	Primary motor cortex
MEP	Motor-evoked potential
MNS	Mirror Neuron System
PD	Personal Distress
PT	Perspective Taking
rmANOVA	Repeated measures analysis of variance
rMT	Resting motor threshold
SD	Standard deviation
SE	Standard Error
TMS	Transcranial magnetic stimulation
