# Supplementary material for: Empathic Dimensions Influence Motor Resonance Magnitude During Transitive but Not Intransitive Action Observation: A Retrospective Investigation
Source: Brain Sci. 2025 Oct 30;15(11):1174. doi: 10.3390/brainsci15111174 (PMC12650464; doi:10.3390/brainsci15111174)
Supplement: Supplementary file 1 [file brainsci-15-01174-s001.zip › brainsci-3931532-supplementary.pdf]

Empathic dimensions predict motor resonance during transitive but not intransitive action observation: A retrospective investigation

# – Supplemental Materials –

Dataset	Type of stimuli showed	Sample size	Sample included	Age (mean ± SD)	Education (mean ± SD)	Edinburgh (mean ± SD)	Stimulator used	rMT (mean ± SD)	rMT determination method	MEP extraction time window (from TMS pulse)	Data taken from
**1**	index finger abduction	18	18 (8 M)	23 ± 2 y	15 ± 2 y	76.9 ± 15.9%	Magstim Rapid 2	60.1 ± 9.7%	5 MEPs out of 10	5-80 ms	[1]
**2**		20	16 (7 M)	22 ± 4 y	15 ± 2 y	74.4 ± 15.5%	Magstim Rapid 2	58.8 ± 9.8%	5 MEPs out of 10	5-80 ms	[1]
**3**		23	22 (10 M)	24 ± 3 y	16 ± 2 y	77.7 ± 14%	Nexstim Eximia	38.2 ± 6.6%	5 MEPs out of 10	15-50 ms	[2]
**4**		25	24 (10 M)	24 ± 2 y	17 ± 2 y	78.1 ± 12.3%	Nexstim Eximia	42.8 ± 8.2%	PEST	5-60 ms	[3]
**5**	whole-hand grasping	35	34 (14 M)	22 ± 2 y	15 ± 2 y	81.8 ± 15.4%	Magstim Rapid 2	60.4 ± 9.5%	PEST	5-60 ms	[4]
**6**		22	22 (9 M)	25 ± 4 y	16 ± 3 y	85.9 ± 11.8%	Magstim Rapid 2	58.9 ± 8.3%	PEST	5-60 ms	pilot data for [4]
**7**		24	24 (10 M)	23 ± 2 y	14 ± 2 y	83.7 ± 15.8%	Nexstim Eximia	44 ± 8.9%	PEST	5-60 ms	unpublished data

Supplemental Table S1. Sample size composition in the seven datasets aggregated in the work.

Condition	FDI (mean ± SE)		
	rest trials	***action trials***	***paired sample t-test***
Intransitive single-digit abduction	1648 ± 96 µV	1837 ± 102 µV	t_{79} = -8.6, p < .001, d = -.96
Intransitive grasping	1676 ± 147 µV	1851 ± 150 µV	t_{79} = -4.76, p < .001, d = -.53
Object-directed grasping	1628 ± 139 µV	1734 ± 141 µV	t_{79} = -2.99, p = .004, d = -.33
Social-directed grasping	1609 ± 127 µV	1728 ± 138 µV	t_{79} = -4.13, p < .001, d = -.46

Supplemental Table S2. Mean ± standard error (SE) of the raw MEPs recorded from FDI in the different experimental conditions. Paired samples t-tests between rest and action trials are also reported.

Condition	ADM (mean ± SE)		
	rest trials	***action trials***	***paired sample t-test***
Intransitive single-digit abduction	1060 ± 87 µV	1114 ± 93 µV	t_{79} = -2.41, p = .077, d = -.28
Intransitive grasping	1100 ± 109 µV	1119 ± 108 µV	t_{79} = -.75, p = .451, d = -.09
Object-directed grasping	1084 ± 104 µV	1085 ± 103 µV	t_{79} = -.07, p = .947, d < .01
Social-directed grasping	1029 ± 91 µV	1033 ± 92 µV	t_{79} = -.15, p = .877, d = .02

Supplemental Table S3. Mean ± standard error (SE) of the raw MEPs recorded from ADM in the different experimental conditions. Paired samples t-tests between rest and action trials are also reported.

**Factor**	$F_{1,158}$	p	η_p^2
**Muscle** (within-subject)	68.44	<.001	.3
**Stimulator model** (between subject)	.12	.727	<.01
**Muscle X Stimulator model**	.37	.533	<.01

Supplemental Table S4. Results from the 'Muscle' (FDI, ADM) X 'Stimulator model' (Magstim, Nexstim) repeated measures ANOVA (rmANOVA) run on MEPs recorded during 'rest' trials to check whether the two stimulators adopted for MEP assessment in the aggregated datasets affect corticospinal excitability during action observation.

Factor	$F_{1,158}$	*p*	η_p^2
**Muscle** (within-subject)	68.65	<.001	.3
**rMT determination procedure** (between subject)	.11	.747	<.01
**Muscle X rMT determination procedure**	.56	.221	<.01

Supplemental Table S5. Results from the 'Muscle' (FDI, ADM) X 'rMT determination procedure' (PEST, 5/10) repeated measures ANOVA (rmANOVA) run on MEPs recorded during 'rest' trials to check whether the two resting motor threshold procedures adopted in the aggregated datasets affect corticospinal excitability during action observation.

IRI scale	$F_{6,159}$	*p*	η_p^2
**PT**	1.55	.166	.06
**FS**	.97	.449	.04
**EC**	.89	.506	.03
**PD**	.55	.769	.02

Supplemental Table S6. Results from the one-way ANOVAs run to check whether the IRI scores in the samples of the studies we aggregated were comparable. A one-way ANOVA with the 7-level factor 'Dataset' was performed for each IRI subscale (PT, FS, EC, PD).

**Condition**	**Predictors**	**model fit**		
		F	***p***	R^2_{adj}
**Intransitive single-digit abduction**	PT, FS, PD, EC	.88	.479	< .01
	cognitive empathy, affective empathy	1.27	.288	.01

Supplemental Table S7. Results from the multiple linear regression models run to check whether the IRI scores (i.e., the 4 subscales and 2 aggregated constructs) are predictive of ADM *motor resonance index* during the observation of intransitive index finger abduction movements.

**Condition**	**Predictors**	**model fit**		
		F	***p***	R^2_{adj}

**Intransitive grasping**	PT, FS, EC, PD	1.3	.276	.02
	cognitive empathy, affective empathy	1.01	.346	< .01
**object-directed grasping**	PT, FS, EC, PD	.37	.83	< .01
	cognitive empathy, affective empathy	.58	.561	< .01
**social-directed grasping**	PT, FS, EC, PD	.31	.87	< .01
	cognitive empathy, affective empathy	.36	.7	< .01

Supplemental Table S8. Results from the multiple linear regression models run to check whether the IRI scores (i.e., the 4 subscales and 2 aggregated constructs) are predictive of ADM *motor resonance index* during the observation of grasping movements.

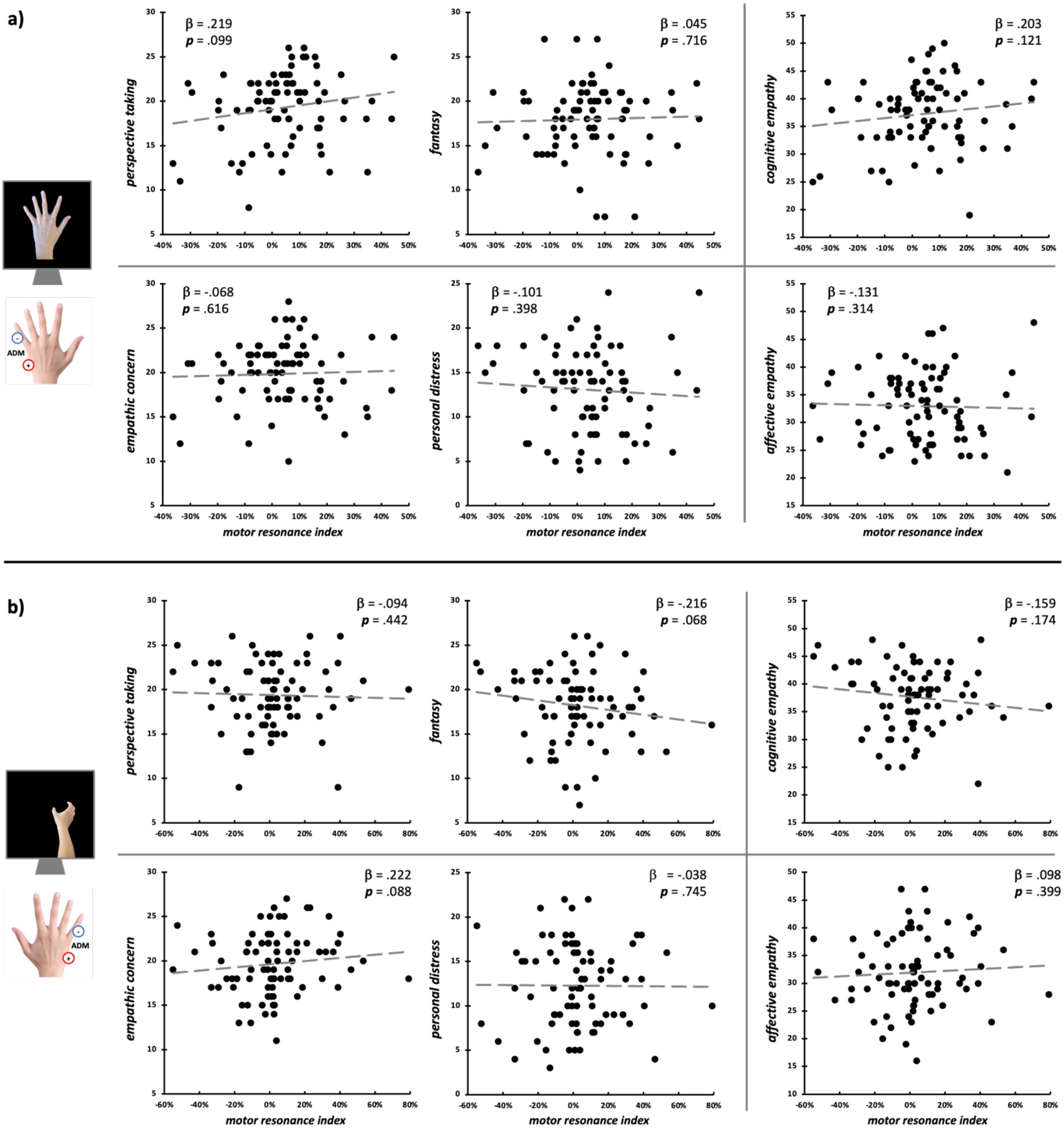

Supplemental Figure S1. Scatterplot between (ADM) *motor resonance index* for intransitive movements (**a**: index finger; **b**: grasping) and IRI scores (left panels: four subscales – PT, FS, EC, PD; right panels: cognitive and affective empathy constructs). Standardized multiple regression coefficients and p-values are reported.

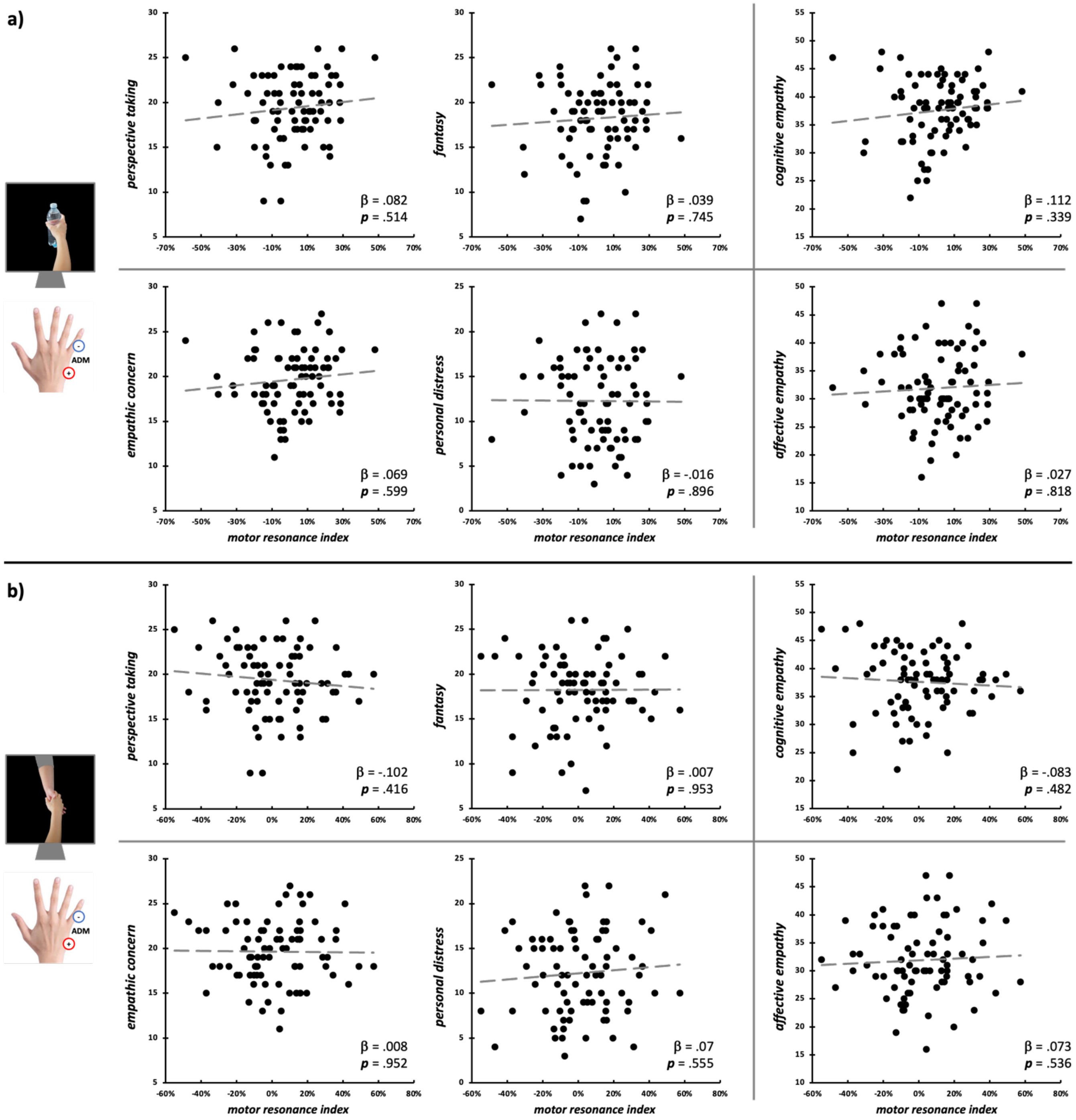

Supplemental Figure S2. Scatterplot between (ADM) *motor resonance index* for transitive grasping movements (**a**: object-directed grasping; **b**: social-directed grasping) and IRI scores (left panels: four subscales – PT, FS, EC, PD; right panels: cognitive and affective empathy constructs). Standardized multiple regression coefficients and p-values are reported.

REFERENCES

1. Guidali, G.; Carneiro, M.I.S.; Bolognini, N. Paired Associative Stimulation Drives the Emergence of Motor Resonance. *Brain Stimul* 2020, *13*, 627–636, doi:10.1016/j.brs.2020.01.017.
2. Guidali, G.; Picardi, M.; Gramegna, C.; Bolognini, N. Modulating Motor Resonance with Paired Associative Stimulation: Neurophysiological and Behavioral Outcomes. *Cortex* 2023, *163*, 139–153, doi:10.1016/j.cortex.2023.03.006.
3. Guidali, G.; Arrigoni, E.; Bolognini, N.; Pisoni, A. M1 Large-Scale Network Dynamics Support Human Motor Resonance and Its Plastic Reshaping. *Neuroimage* 2025, *308*, 121082, doi:10.1016/j.neuroimage.2025.121082.
4. Guidali, G.; Picardi, M.; Franca, M.; Caronni, A.; Bolognini, N. The Social Relevance and the Temporal Constraints of Motor Resonance in Humans. *Sci Rep* 2023, *13*, 15933, doi:10.1038/s41598-023-43227-2.
